# Long Vax in the Eye: Long Post-COVID Vaccination Syndrome Presenting with Frosted Branch Angiitis

**DOI:** 10.3390/diseases12020036

**Published:** 2024-02-09

**Authors:** Koju Kamoi, Kyoko Ohno-Matsui

**Affiliations:** Department of Ophthalmology & Visual Science, Graduate School of Medical and Dental Sciences, Tokyo Medical and Dental University, Tokyo 113-8510, Japan

**Keywords:** mRNA COVID-19 vaccine, long post-COVID vaccination syndrome, long vax, ocular inflammation, frosted branch angiitis

## Abstract

mRNA COVID-19 vaccines have been reported as protecting against COVID-19 and reducing its severity, and we have recognized post-vaccination symptoms recently. This research investigates the clinical trajectories of ocular disorders in a 51-year-old female who received a second dose of the BNT162b2 (Pfizer-BioNTech) mRNA COVID-19 vaccine. Exhibiting fever and blurred vision within 24 h post-vaccination, with progressive blurry vision over two months, she underwent in-depth ophthalmologic examinations, revealing intraocular cellular infiltration in anterior chamber, vitreous opacity, and frosted branch angiitis in both eyes. Comprehensive evaluations, including systemic workups as well as ocular and blood specimen analyses, excluded autoimmune and infectious etiologies, consolidating the diagnosis of vaccine-induced ocular inflammation. Despite adherence to prevailing therapeutic protocols, her condition showed no significant improvement over 18 months, pointing to a possible long post-COVID vaccination syndrome. Such persistent sequelae underscore the need for detailed studies to discern the interactions between vaccine-induced immune responses and the development of post-vaccination sequelae. Continual documentation of patients with long post-COVID vaccination syndrome is now essential to better understand the vaccine’s immunological effects, aiding in improving global vaccination strategies.

## 1. Introduction

As the global community continues to confront the challenges posed by the Coronavirus disease 2019 (COVID-19) pandemic, the role of mRNA COVID-19 vaccines has been pivotal. These vaccines, a cornerstone in the fight against the pandemic, have proven their efficacy in protecting against the virus and mitigating the severity of the disease [1]. In Japan, a country significantly impacted by the severe acute respiratory syndrome coronavirus-2 (SARS-CoV-2), the adoption of these vaccines has been particularly strategic and effective. With more than 33 million individuals infected, and nearly 75 thousand fatalities attributed to COVID-19, the nation’s commitment to vaccination has been a critical element in its public health response. Japan’s successful implementation of a vaccination strategy, achieving approximately 80% coverage of the second vaccine dose among its population, stands as a testament to the effectiveness of these vaccines in controlling the spread of COVID-19 [2].

The mechanism by which mRNA COVID-19 vaccines operate involves inducing a robust and sustained enhancement of the immune system [3]. This process effectively creates a protective barrier against the virus, equipping the body with a heightened defense against infection. The vaccines’ ability to provide increased protection against severe COVID-19-related outcomes, including those caused by SARS-CoV-2 variants, is a significant achievement in medical science.

However, the immune response elicited by these vaccines, while largely beneficial, can sometimes lead to unintended consequences. Among these is the potential disruption of immune homeostasis, which can manifest as inflammation in various body structures, including the eyes [4,5]. In fact, we reported an association between multiple prevalent vaccines and several forms of ocular inflammation, such as anterior, intermediate, and posterior uveitis [6]. Corticosteroids were effective as a treatment, yet it is crucial to note that full recovery was not achieved in half of the cases, pointing to potential long-lasting effects of these vaccines [6].

This particular aspect of the vaccine’s impact is becoming increasingly important as the medical community observes a rise in post-vaccination symptoms that bear resemblance to Long COVID, now increasingly referred to as Long Vax [7,8]. Understanding the specific immunological pathways activated by mRNA vaccines is, therefore, becoming a critical area of study, especially in the context of these prolonged symptoms.

## 2. Case Presentation

Our detailed investigation into post-vaccination complications centers around a 51-year-old female patient, highlighting a significant case in the context of mRNA COVID-19 vaccination and its potential ocular side effects. Her medical history includes surgeries for ovarian cystoma, uterine fibroid, and colon polyp. Additionally, she has been diagnosed with hypertension and allergic rhinitis, but she has no specific systemic disease nor a family history of inflammatory diseases such as rheumatoid arthritis. The patient received the BNT162b2 (Pfizer-BioNTech) mRNA COVID-19 vaccine, with the first dose administered smoothly without any adverse effects. However, the situation took a turn following the administration of the second dose. Within a span of 24 h, she developed symptoms including fever and blurry vision in both eyes, a development that necessitated immediate medical management with acetaminophen at a dosage of 1500 mg/day. Although the fever subsided within two days, the blurry vision not only persisted, but also worsened progressively over the following two months, raising concerns and prompting a thorough ophthalmologic examination.

**Figure 1:**
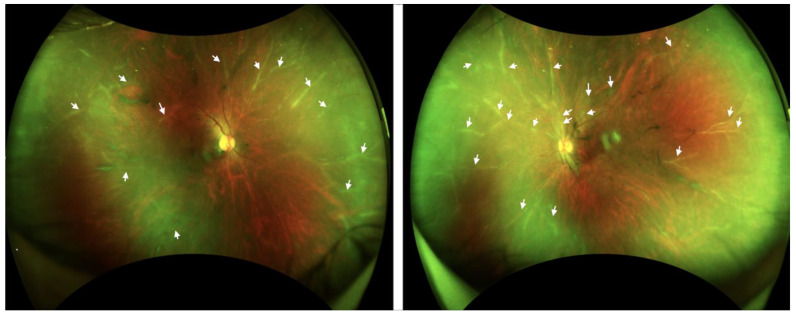
Wide-field fundus imaging. A diffuse perivascular sheath-like frosted branch in the retinal vessels (arrows) and vitreous opacity was detected in both eyes.

Her best-corrected visual acuity (BCVA) was 1.2 in the right eye, and 0.5 in the left eye. The intraocular pressure (IOP) was 20 mm Hg in both her eyes. During the detailed ophthalmologic examination, several key findings were noted. A slit-lamp examination of the patient’s eyes revealed cellular infiltrates present in the anterior chamber and the vitreous of both eyes. This was a critical observation, as such infiltrates can be indicative of underlying inflammatory processes. Further examination of the fundus revealed additional concerning features: there was noticeable vitreous opacity and the presence of diffuse perivascular sheath-like frosted branches in the retinal vessels, a characteristic described in Figure 1 of our report. Macular edema was absent in both eyes.

**Figure 2:**
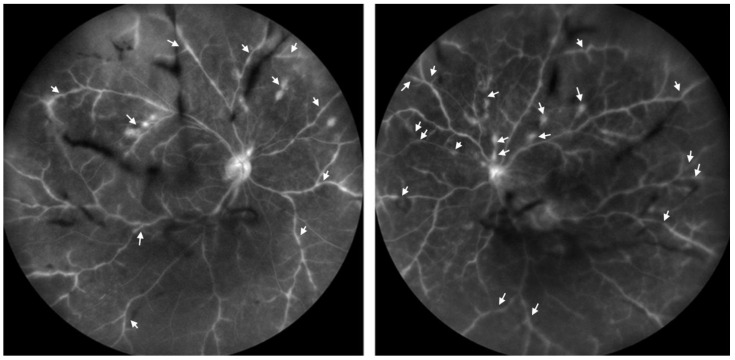
Wide-field fluorescein angiography. Staining and leakage of dye along the retinal vascular sheath (arrows) was identified in both eyes, with no signs of occlusion or stasis in the retinal vessels. The vitreous opacities were identified as black shadows.

Fluorescein angiography, a diagnostic procedure used to visualize the retinal vasculature, showed staining and dye leakage along the retinal vascular sheath. Importantly, there were no signs of occlusion or stasis, as documented in Figure 2, ruling out certain other vascular disorders.

To explore the possibility of underlying systemic conditions or autoimmune disorders that could explain these ocular findings, a comprehensive systemic and blood work-up was conducted. This extensive investigation conclusively ruled out a variety of autoimmune afflictions, including sarcoidosis, lupus erythematosus, antineutrophil cytoplasmic antibody (ANCA)-associated vasculitis, Behcet’s disease, giant cell arteritis, and eosinophilic granulomatosis with polyangiitis. Detailed blood assays were performed, covering a wide range of markers and antibodies, including angiotensin-converting enzyme, lysozyme, anti-nuclear antibody, anti-DNA antibody, anti-cardiolipin antibody-IgG, anti-β2-glycoprotein I antibody, anti-cyclic citrullinated peptide antibody, matrix metalloproteinase-3, anti-CCP antibody, anti-SSA antibody, anti-SSB antibody, anti-Sc1-70 antibody, anti-ARS antibody, mitochondrial antibody, PR3-ANCA, and MPO-ANCA, KL-6, and globulin, along with complement components C3 and C4. All test results were within the normal range. Radiographic studies and clinical examinations were thorough and confirmed the absence of systemic abnormalities, joint swelling, Gottron’s sign, or any history of recurrent oral and genital ulcers.

Simultaneously, screening for infectious diseases was carried out, considering the differential diagnosis of infectious uveitis. Tests for syphilis, mycobacterium tuberculosis, cytomegalovirus (CMV), human immunodeficiency virus (HIV), human T-lymphotropic virus type 1 (HTLV-1), and fungal infections were conducted. All results were negative, including specific tests such as treponema pallidum hemagglutination, rapid plasma reagin card agglutination, interferon gamma release assay, CMV antigenemia, anti-HIV antibody, anti-HTLV-1 antibody, and β-D glucan. Furthermore, vascular infarction analysis was performed, including prothrombin time (PT), activated partial thromboplastin time (APTT), fibrinogen, and lipid panel, all of which were within normal limits.

In addition to these tests, ocular specimen investigations were also included. Utilizing multiplex polymerase chain reaction (PCR) of the aqueous humor, we evaluated for viral infections such as HSV-1 and -2, VZV, Epstein–Barr virus, CMV, and human herpesvirus 6–8, and parasitic infections like toxoplasma. Remarkably, all these potential causative agents for frosted branch angiitis were ruled out, as noted in references [9,10] of our report.

Given the comprehensive diagnostic findings, and the exclusion of other potential causes, we considered the possibility that the presentation of frosted branch angiitis could be an adverse reaction to the mRNA COVID-19 vaccine. Therefore, a prevailing therapeutic regimen typically utilized for non-infectious uveitis was initiated [11]. The treatment plan included systemic prednisolone therapy at a dosage of 0.5 mg/kg/day, and 0.1% betamethasone eye drops as the primary treatment agents. However, despite a three-month course of this steroid therapy, the patient’s symptoms showed no improvement. This lack of response necessitated further intervention in the patient’s treatment plan.

Subsequently, Methotrexate, an immunosuppressive drug often used in the treatment of various inflammatory disorders, was introduced at an initial dose of 6 mg/week. Due to the persistent nature of the patient’s symptoms, the dosage was later escalated to 12 mg/week. However, even with these therapeutic adjustments, the frosted branch angiitis continued to persist over the next six months. This led to the introduction of adalimumab, an anti-TNF-alpha monoclonal antibody, into the treatment regimen. Initially administered at 80 mg, the dosage was later adjusted to 40 mg biweekly.

**Figure 3:**
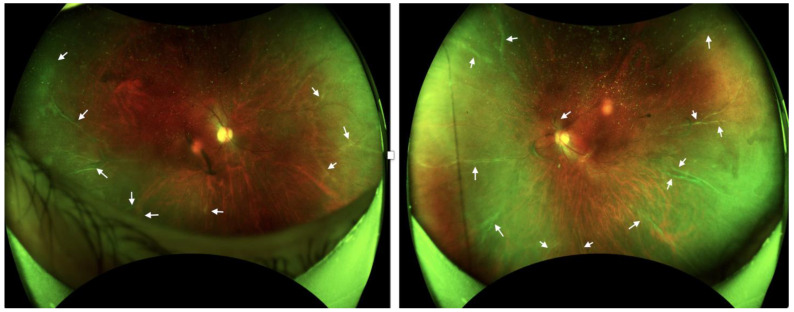
Wide-field fundus imaging. A diffuse perivascular sheath-like frosted branch in the retinal vessels (arrows) was still observed in both eyes.

**Figure 4:**
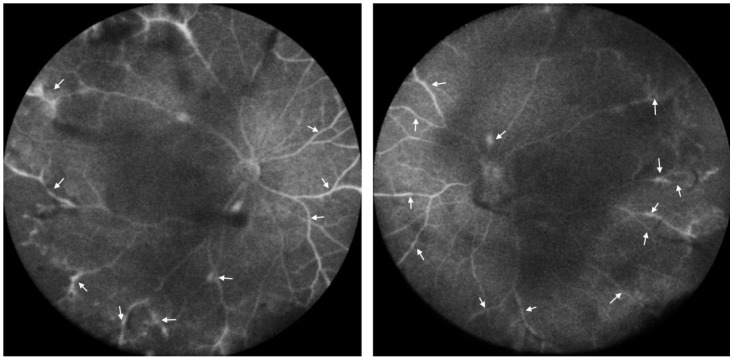
Wide-field fluorescein angiography. Staining and leakage of dye along the retinal vascular sheath (arrows) was still identified in both eyes.

Continuous monitoring over an 18-month period revealed no significant improvement in the patient’s condition, despite these extensive and varied therapeutic interventions. Her BCVA was 1.0 in the right eye, and 0.8 in the left eye. The IOP was 18 mm Hg in both her eyes. The patient’s persistence of frosted branch angiitis, as illustrated in Figure 3 and Figure 4, despite treatment with steroids, methotrexate, and adalimumab, presents a significant clinical challenge and underscores the complexities associated with the management of vaccine-induced immune responses.

## 3. Discussion

The introduction and widespread use of mRNA COVID-19 vaccines, such as the BNT162b2 (Pfizer-BioNTech) vaccine, represent a significant milestone in the global fight against the COVID-19 pandemic. These vaccines have been instrumental in reducing the severity of the disease and providing essential protection against the virus. However, the emergence of post-vaccination complications, most notably the phenomenon of “Long Vax” syndrome, presents a new and intricate challenge that necessitates extensive research and understanding [8].

Long Vax syndrome, characterized by persistent and sometimes debilitating post-vaccination symptoms, has brought to light the potential long-term effects of mRNA vaccines. The similarities between the manifestations of this syndrome and Long COVID underscore the complex and multifaceted nature of the body’s response to these vaccines [12,13]. This new clinical entity highlights the urgent need for thorough investigation into the immunological mechanisms behind vaccine-induced reactions.

Our case study, highlighting the rare occurrence of frosted branch angiitis in a patient following mRNA COVID-19 vaccination, exemplifies the complexities inherent in diagnosing and managing these novel post-vaccination phenomena. The persistence of the patient’s symptoms, despite multiple therapeutic interventions, points to a nuanced interaction between the vaccine components, the individual’s immune response, and possible genetic predispositions. It emphasizes the critical importance of ongoing vigilance in vaccine safety monitoring and the necessity for personalized approaches in vaccine administration, particularly in light of the unique challenges posed by Long Vax syndrome.

As the global medical community continues to navigate the intricacies of the COVID-19 pandemic, the phenomenon of Long Vax provides a poignant reminder of the ever-evolving nature of this public health crisis. It highlights the need for continuous research, robust post-vaccination surveillance, and a deeper understanding of the individualized nature of vaccine responses. These efforts are critical not only for managing the immediate effects of vaccination, but also for shaping future vaccine development and administration strategies, ensuring their safety and efficacy.

Frosted branch angiitis, first reported in 1976, is a rare retinal vasculitis characterized by a distinctive frosted appearance of the perivascular exudate. It predominantly affects young, healthy individuals with a bimodal age distribution, peaking in childhood and the third decade. It has a higher prevalence in females, and is more common in the Japanese population. Typical symptoms include subacute visual loss, floaters, and photopsiae, often with bilateral involvement. Frosted branch angiitis’ fundal appearance is striking, with widespread and bilateral retinal vasculitis. The condition may include mild to moderate iritis, vitritis, retinal edema, and occasionally papillitis. Most patients respond well to systemic steroids, with rapid resolution and good visual recovery, although some cases take months to recover. Recurrences are rare, but complications can include macular scarring, retinal occlusion, and optic disc atrophy [14].

The cause of frosted branch angiitis remains unknown, though it is hypothesized to be a hypersensitivity reaction to various infective agents, initiating via an immune-complex deposition pathway. Other causes of retinal vasculitis should be excluded, including viral retinitis and systemic conditions like sarcoidosis and Behcet’s disease. In some instances, frosted branch angiitis has been linked to systemic conditions or intraocular infections, and it can occasionally be secondary to infections like CMV or HSV [14]. Recently, several cases of frosted branch angiitis following mRNA COVID-19 vaccination have been reported [15,16,17,18], suggesting that the vaccine might be a new trigger for frosted branch angiitis. However, instances of long-lasting frosted branch angiitis, or ‘Long Vax’ syndrome, characterized by prolonged ocular inflammation after mRNA COVID-19 vaccination, were previously unknown and highlight a new area of interest in vaccine-related adverse effects.

Our in-depth exploration of this syndrome revolves around the case of a 51-year-old female patient who developed ocular inflammation, specifically frosted branch angiitis, after receiving her second dose of an mRNA COVID-19 vaccine. This case is noteworthy due to its rarity and the potential insights it offers into vaccine-induced immune responses. The patient’s experience, which started with fever and blurry vision and progressed to persistent and worsening visual impairment, underscores the complexity of vaccine-related side effects. This progression necessitated a comprehensive diagnostic approach, encompassing extensive evaluations to exclude autoimmune disorders and infectious diseases. The exhaustive diagnostic process involved systemic and blood work-ups [10], which meticulously ruled out various autoimmune disorders and aligned clinical suspicions with a vaccine-induced cause. This hypothesis was further supported by extensive screenings for infectious diseases, which were negative, effectively excluding common infectious etiologies of uveitis.

The clinical findings from ocular examinations were crucial. The slit-lamp and fundus examinations, coupled with fluorescein angiography, painted a vivid picture of frosted branch angiitis. This manifestation of retinal vasculitis, though previously observed in other systemic conditions, presented unique challenges in the context of post-vaccination. The persistence of symptoms, despite treatment with systemic prednisolone, Methotrexate, and adalimumab, underscored the resistant nature of this condition.

The pathogenesis of vaccine-associated uveitis following mRNA COVID-19 vaccination remains a subject of intense research and debate. The molecular mimicry hypothesis posits that vaccine-induced antibodies may erroneously target ocular tissues due to structural similarities with certain viral proteins. This theory is bolstered by the observed homology between some viral and human proteins. In the context of COVID-19 vaccines, designed around the spike protein’s genetic code, such cross-reactivity could conceivably lead to ocular inflammation [19].

Another mechanism under scrutiny is immune complex deposition resulting from Type III hypersensitivity reactions. Here, the antigen–antibody complexes formed post-vaccination may not be effectively cleared, leading to their deposition in tissues such as the eye and consequent inflammation. The elevated levels of immune complexes in the serum and ocular fluids of patients with uveitis lend credence to this hypothesis.

The potential role of vaccine adjuvants in inducing uveitis is also a critical area for investigation. Adjuvants, intended to enhance the immune response to the vaccine antigen, may in some instances trigger autoinflammatory and autoimmune reactions, particularly in genetically susceptible individuals. This phenomenon, known as Autoimmune/Inflammatory Syndrome Induced by Adjuvants (ASIA) or Shoenfeld’s syndrome, has been noted in various vaccinations [20,21]. The employment of lipid nanoparticles in mRNA COVID-19 vaccines as adjuvant-like substances raises questions about their potential involvement in ocular inflammation.

The genetic predisposition of individuals to adverse vaccine reactions is another vital area of study. Various HLA haplotypes have been associated with different autoimmune conditions, including those triggered by vaccines. Investigating these genetic factors is critical for understanding individual susceptibilities to adverse vaccine reactions, and could inform more tailored vaccine administration strategies [22].

This case study, with its unique presentation of frosted branch angiitis post-mRNA vaccination, underscores the necessity for a more profound understanding of these complex immunological mechanisms. The patient’s persistent symptoms, despite comprehensive treatment, indicate a multifaceted interaction between vaccine components, the immune system, and potential genetic factors. This case not only emphasizes the importance of continued vigilance in monitoring vaccine safety, but also the need for personalized approaches in vaccine administration.

The emergence of Long Vax syndrome, as exemplified by persistent manifestations such as frosted branch angiitis, necessitates sustained and comprehensive research. This condition shines a light on the complexity of the body’s response to mRNA vaccines and raises critical questions about the mechanisms of vaccine-induced immune responses. Investigating the roles of molecular mimicry, immune complex deposition, and the influence of vaccine adjuvants is essential for understanding these responses, especially in light of individual genetic predispositions [5,23].

Furthermore, this case highlights the crucial role of global collaboration in vaccine safety monitoring. The rapid development and deployment of mRNA COVID-19 vaccines were a testament to international cooperation. Similarly, addressing the challenges posed by Long Vax syndrome requires a concerted effort from the global health community. Sharing data, experiences, and research findings across borders can enhance our understanding of vaccine-induced complications and lead to more effective strategies for prevention and management. This global approach also extends to public health policies and education. Increasing awareness about potential vaccine side effects, while balancing this information with the benefits of vaccination, is essential for informed decision-making by the public. Health authorities and medical professionals must work together to communicate risks effectively and provide clear guidance on managing any adverse reactions.

The insights gained from these investigations are not only crucial for managing immediate effects, but also for influencing the future development and administration of vaccines. Strengthening vaccine safety monitoring systems and ensuring robust post-vaccination surveillance are key to maintaining public trust in vaccination programs [24].

## 4. Conclusions

The case of Long Vax syndrome, with its specific challenges and implications, serves as a catalyst for advancing our understanding of vaccine safety and efficacy. It calls for an integrated approach that combines clinical vigilance, scientific inquiry, and international collaboration. As we continue to navigate the complexities of the COVID-19 pandemic, the lessons learned from cases like this will be invaluable in shaping resilient and responsive health systems, capable of addressing both current and future vaccine-related challenges.

## Data Availability

All data relevant to the study are included in the article.
